# Apoptosis as Driver of Therapy-Induced Cancer Repopulation and Acquired Cell-Resistance (CRAC): A Simple In Vitro Model of Phoenix Rising in Prostate Cancer

**DOI:** 10.3390/ijms23031152

**Published:** 2022-01-21

**Authors:** Francesca Corsi, Francesco Capradossi, Andrea Pelliccia, Stefania Briganti, Emanuele Bruni, Enrico Traversa, Francesco Torino, Albrecht Reichle, Lina Ghibelli

**Affiliations:** 1 Department of Biology, University of Rome “Tor Vergata”, 00133 Rome, Italy; francesco.capradossi@uniroma2.it (F.C.); andreapelliccia64@alumni.uniroma2.ue (A.P.); emanuele.bruni@uniroma2.it (E.B.); 2 PhD Program in Evolutionary Biology and Ecology, Department of Biology, University of Rome “Tor Vergata”, 00133 Rome, Italy; 3 Department of Chemical Science and Technologies, University of Rome “Tor Vergata”, 00133 Rome, Italy; traversa.enrico@gmail.com; 4 Cutaneous Physiopathology and Integrated Center of Metabolomics Research, San Gallicano Dermatological Institute, IRCCS, 00144 Rome, Italy; stefania.briganti@ifo.gov.it; 5 School of Materials and Energy, University of Electronic Science and Technology of China, Chengdu 610056, China; 6 Department of Systems Medicine, Medical Oncology, University of Rome Tor Vergata, 00133 Rome, Italy; torino@med.uniroma2.it; 7 Department of Internal Medicine III, Hematology and Oncology, University Hospital of Regensburg, 93053 Regensburg, Germany; albrecht.reichle@klinik.uni-regensburg.de

**Keywords:** caspase-3, chemoresistance, XIAP, PC3, LNCaP, PGE-2, EMT

## Abstract

Apoptotic cells stimulate compensatory proliferation through the caspase-3-cPLA-2-COX-2-PGE-2-STAT3 Phoenix Rising pathway as a healing process in normal tissues. Phoenix Rising is however usurped in cancer, potentially nullifying pro-apoptotic therapies. Cytotoxic therapies also promote cancer cell plasticity through epigenetic reprogramming, leading to epithelial-to-mesenchymal-transition (EMT), chemo-resistance and tumor progression. We explored the relationship between such scenarios, setting-up an innovative, straightforward one-pot in vitro model of therapy-induced prostate cancer repopulation. Cancer (castration-resistant PC3 and androgen-sensitive LNCaP), or normal (RWPE-1) prostate cells, are treated with etoposide and left recovering for 18 days. After a robust apoptotic phase, PC3 setup a coordinate tissue-like response, repopulating and acquiring EMT and chemo-resistance; repopulation occurs via Phoenix Rising, being dependent on high PGE-2 levels achieved through caspase-3-promoted signaling; epigenetic inhibitors interrupt Phoenix Rising after PGE-2, preventing repopulation. Instead, RWPE-1 repopulate via Phoenix Rising without reprogramming, EMT or chemo-resistance, indicating that only cancer cells require reprogramming to complete Phoenix Rising. Intriguingly, LNCaP stop Phoenix-Rising after PGE-2, failing repopulating, suggesting that the propensity to engage/complete Phoenix Rising may influence the outcome of pro-apoptotic therapies. Concluding, we established a reliable system where to study prostate cancer repopulation, showing that epigenetic reprogramming assists Phoenix Rising to promote post-therapy cancer repopulation and acquired cell-resistance (CRAC).

## 1. Introduction

Cytotoxic anticancer therapies promote damage-induced apoptosis in cancer cells, reducing tumor mass; however, the disease often relapses or progresses due to repopulation of the depleted cancer tissues by the cells surviving the treatment [1]. During this process, cells may increase in malignancy, metastasize and acquire drug resistance, suggesting that therapy itself favors tumor progression [2,3,4]. Such adverse effects of the therapies are generally attributed to the selective survival of those cancer cells carrying the most aggressive genetic mutations, thereby evading apoptosis [5]. Recently, however, this linear view was challenged by evidence showing a more complex scenario. Indeed, cancer tissues wounded by the therapy react by setting-up a series of aberrant responses via non-genetic mechanisms, activating pathways aiming at tissue repair and repopulation, but at the same time promoting tumor immune escape, evasion from primary site, genetic instability and acquired resistance [6,7,8,9,10,11,12]. This implies that cancer cells, rather than being passive subjects of natural selection, would play an active signaling role in setting up therapy-promoted tumor progression. 

At least two such scenarios have been coherently described, which participate in the reorganization of the wounded cancer tissues. 

On one side, cytotoxic therapies promote cell plasticity via epigenetic reprogramming [6]: cells surviving the cytotoxic treatments activate preset signaling packages, promoting the coordinated expression of cell resistance, cell proliferation and cell motility genes. Various homeostatic pathways participate in this process, entangling highly branched signaling axes. Among them, epithelial-to-mesenchymal-transition (EMT) is a key pleiotropic process usurped from embryonal development [7], where cells lose epithelial adhesion, thus facilitating evasion from the tumor site [13]. EMT is typically accompanied by other features that cooperate to provide a selective advantage to the cells, consisting in the acquisition of directional motion [9], increased cell survival [10], loss of anoikis [11], loss of cell integrity checkpoints, and acquisition of genetic instability [12]. In such processes, which are very well documented, surviving cells actively organize the repopulation; this explains how cytotoxic therapies may promote tumor progression through *ad hoc* reprogramming survivors’ gene expression in a pro-malignant way, rather than selecting randomly occurring genetic mutations. 

On the other side, it is emerging that therapy-hit apoptotic cells emit paracrine replicative signals, promoting compensatory proliferation among surviving cells through the caspase-3 → cytosolic phospholipase A2 (cPLA2) → cyclooxygenase-2 (COX-2) → prostaglandin E2 (PGE-2) → EP4/2 → STAT3 axis [14,15,16]. In this case, the active role is paradoxically played by the dying cells themselves, which promote tissue repopulation; hence, the term “Phoenix Rising”, from the mythical bird re-born from its own ashes [17]. Apoptosis, in this view, is not the end-point of the therapies, but a key turning point of the side effects triggered by pro-apoptotic therapies [18], with the result that the initial benefits of tumor shrinkage due to apoptosis are overwhelmed by a successive exaggerated repopulation. Though usurped by cancer tissues, Phoenix Rising is in fact an important homeostatic mechanism responsible for regeneration all over the animal kingdom [19], including liver regeneration in humans [20]. Caspase-3, the protease that coordinates cell dismantling during apoptosis, plays the counter-intuitive role of triggering the repopulation of injured tissues, coordinating cell death with proliferation, thus allowing restoration of a functional organ size. This indicates that repopulation is not an automatic process but necessitates a proper signaling in order to take place; importantly, such a notion hardly emerges from the epigenetic reprogramming studies related to cancer and EMT.

The studies on compensatory proliferation indicate that Phoenix Rising drives post-damage repopulation in both normal and cancer tissues [21]. However, as pointed out above, cancer tissues, unlike normal ones, repopulate with increased malignancy. Is there a link between repopulation and the epigenetic reprogramming leading to EMT, that may explain why Phoenix Rising differs in normal vs. cancer tissues? To explore this issue, we investigated the initial events occurring during post-therapy repopulation in cancer vs. normal tissue, by setting up a straightforward one-pot in vitro model mimicking a mono-stratified epithelium such as prostate’s. We found that this simple, purely epithelial model, treated with the chemotherapeutic drug etoposide, effectively reproduces the complexity of the wounded tissue’s response to damage. This allowed us to show that post-therapy repopulation occurs via Phoenix Rising in normal and cancer prostate cells, but in the latter, epigenetic reprogramming is required to complete the pathway.

## 2. Results

### 2.1. Modeling Etoposide-Induced Cancer Repopulation and Acquired Cell Resistance in PC3

PC3 cells are derived from an androgen-insensitive, castration-resistant high-grade PCa, displaying neuroendocrine features [22]; in such cases, patients are often treated with chemotherapy, including the topoisomerase II poison etoposide [23]. We use here the etoposide/PC3 system to explore the series of events occurring in response to a clinically relevant chemotherapy treatment.

**Figure 1:**
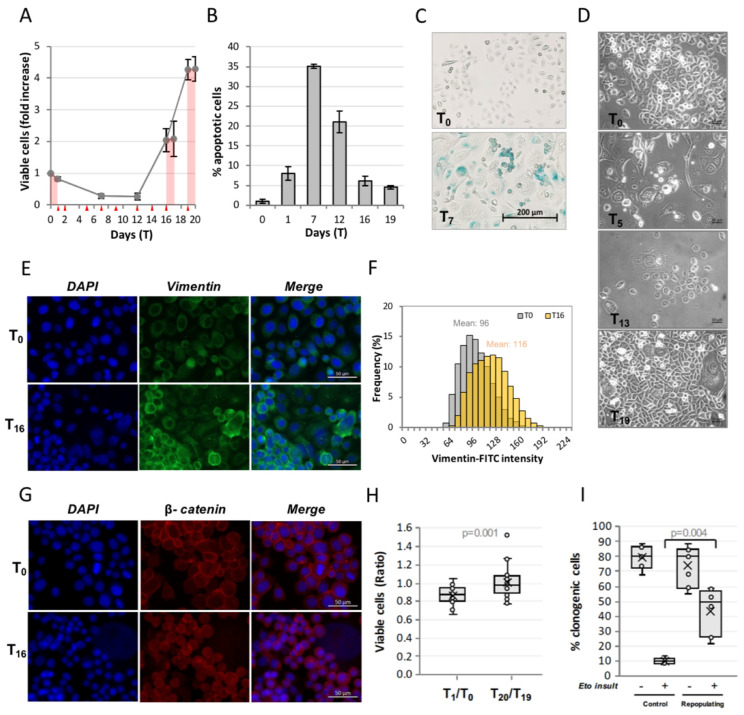
**Modeling etoposide-induced cancer repopulation and acquired cell resistance in PC3.** Etoposide-treated PC3 cells undergo repopulation after the degenerative phase. (**A**) PC3 cells were treated with 50 µM etoposide for 24 h and allowed to recover for 18 days. The profile shows the kinetics of viable (trypan blue-excluding) cell number expressed as Fold over T_0_. At day 16 or 19, a second 50 µM etoposide treatment was administered. Pink areas indicate etoposide treatments, red triangles indicate medium change; (**B**) The degenerative phase is characterized by apoptosis: fraction of apoptotic cells quantified by nuclear morphology. All values are the mean of ≥3 independent experiments ± SD; (**C**) Etoposide-treated PC3 undergo senescence: beta-galactosidase staining at T_0_ and T_7_; (**D**) Phase-contrast images of PC3 during the successive phases of CRAC at the indicated time points; (**E**,**F**,**G**) Repopulating cells undergo epithelial-to-mesenchymal transition, strongly overexpressing the EMT-marker vimentin (**E**) and nuclear β-catenin (**G**): fluorescence microscope images (**E**,**G**) and vimentin intensity quantification (**F**) at T_0_ and T_16_. The mean value is reported above each intensity distribution; (**H**) Repopulation implies acquired resistance to a second insult: ratio between cell number before (T_0_ and T_19_) and after (T_1_ and T_20_) the first vs. second etoposide treatment; the second treatment fails to decrease cell number, revealing a loss of efficacy; (**I**) PC3 strongly increase the clonogenic ability after the second vs. the first insult: fraction of clonogenic cells before and after the first (=control) vs. second (=repopulating cells) insult of etoposide; note the 5x increase of clonogenic potential of repopulating vs. untreated cells. Statistical significance was calculated via a Mann–Whitney test: *p*-values are reported at the top of each panel.

Figure 1A shows the evolution of a PC3 culture treated for 24 h with 50 µM etoposide, followed by 18 days of recovery, in terms of the number of viable cells; pink areas indicate treatments with 50 µM etoposide; red triangles indicate medium change; days of treatment are indicated as T_x_, T_0_ being untreated cells.

Cell number decreases during etoposide treatment and is further reduced during the days following drug removal (T_1_ through T_7_); this degenerative phase is characterized by high levels of apoptosis, which peaks at T_7_ (Figure 1B). During this phase, most cells show senescence traits, as revealed by beta-galactosidase staining (Figure 1C). Afterwards, the culture enters a kinetically quiescent phase; however, microscopic monitoring shows that surviving cells are highly active in this period, acquiring different morphologies (Figure 1D): some reach giant size, others emit tubular protrusions, and others produce simil-lamellipodia suggestive of enhanced motion.

At T_12_, cell number begins to increase, accelerating the proliferation rate at T_16_, consistently with a regenerative phase (repopulation). Repopulation starts from a very tiny number of permissive cells, estimated as <0.03% of the surviving cells by visual inspection of the repopulating plates (data not shown). Medium conditioned by repopulating cells is able to accelerate proliferation of untreated PC3 cells (not shown), indicating production of paracrine signaling molecules, possibly responsible for repopulation of etoposide-treated cells.

Repopulating cells totally lose beta-galactosidase staining, which is coherent with the notion that cancer cells can escape from senescence (not shown). Importantly, they undergo EMT, as shown by the strong and stable overexpression of the mesenchymal marker vimentin (Figure 1E,F) and by β-catenin redistribution from the cell membrane to the nucleus (Figure 1G). Importantly, this is associated with a decreased sensitivity to etoposide: on one side, the extent of etoposide-induced cell loss is significantly reduced among repopulating vs. original cells (Figure 1H); on the other, the number of cells able to originate colonies increases by 5-fold after the second vs. first etoposide treatment (Figure 1I).

Overall, these results show that this in vitro model reliably represents a simplified version of wounded cancer pseudo-tissue coping with chemo-induced stress and undergoing EMT in order to repopulate, simultaneously acquiring chemo-resistance. Importantly, this in vitro model of post-therapy cancer repopulation is self-sufficient, including both cells able to emit pro-repopulation signals, and cells able to respond to such signals.

### 2.2. Post-Therapy Repopulation in PC3 Occurs via Phoenix Rising

**Figure 2:**
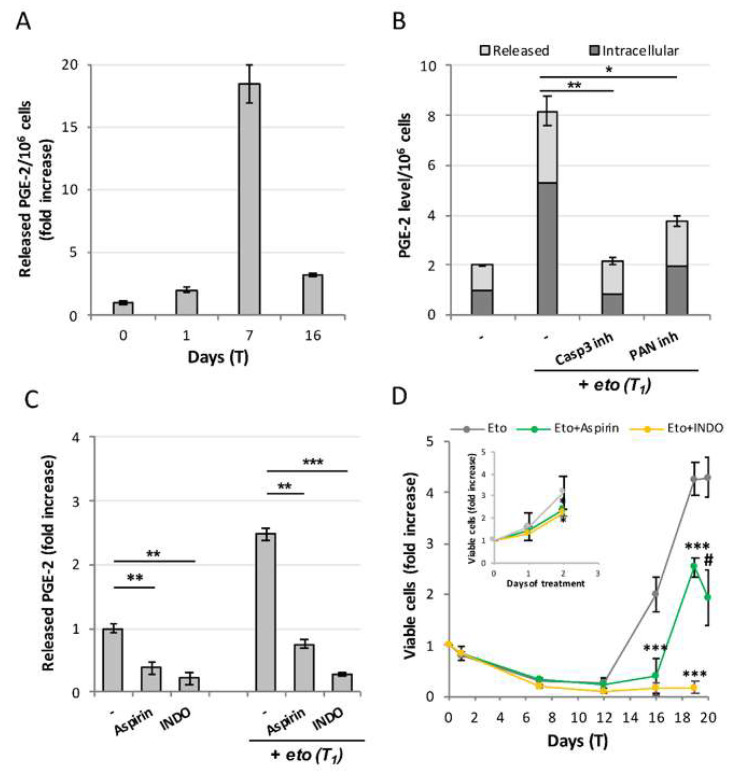
**Repopulation occurs via Phoenix Rising.** (**A**) Time-course of PGE-2 production in etoposide-treated PC3. Concentration of released PGE-2/10^6^ cells expressed as fold increase with respect to control values (65.2 nM); (**B**) Caspase-3 inhibition prevents PGE-2 production: intracellular (dark bars) and released (light bars) PGE-2 concentration values are expressed as fold increase over the relative controls; (**C**) Indomethacin and aspirin prevent PGE-2 production. Values are the mean of ≥3 independent experiments ± SD. Statistical significance was calculated via Student’s T test. ** *p* < 0.01 and *** *p* < 0.001 with respect to controls. (**D**) COX-2 inhibitors affect repopulation in etoposide-treated PC3: kinetics of viable (trypan blue-excluding) cell number in the presence/absence of the COX-2 inhibitors indomethacin (INDO) or aspirin; the inhibitors were added at T_0_ and re-administered at each medium change. Inset: aspirin and INDO slightly affect untreated PC3’s growth rate. Statistical significance was calculated via Student’s T test. * *p* < 0.05, ** *p* < 0.01 and *** *p* < 0.001 refer to the eto group. # *p* = 0.044 refers to T_19_ of the same group.

We explored the mechanisms through which etoposide-treated PC3 repopulate. Figure 2A shows that CRAC implies a very strong production of PGE-2, whose levels reach values up to 18-fold higher over controls, peaking at T_7_ (i.e., concomitantly with the apoptosis peak, see Figure 1B). Strikingly, caspase-3-inhibition prevents PGE-2 overproduction (Figure 2B), both at the intracellular (dark bars) and secreted (light bars) level; the pan-caspase inhibitor z-VAD is slightly less effective. This evidence points to caspase-3 as the main (only?) responsible for increasing PGE-2 synthesis upon etoposide challenge.

To explore the role of PGE-2 in repopulation, we used two standard COX-2 inhibitors, indomethacin and aspirin. Both compounds strongly impair PGE-2 production, already at T_1_ (Figure 2C); indomethacin, which inhibits both COX-1 and COX-2, is slightly more effective than aspirin, which is instead a specific COX-2 inhibitor. Repopulation is totally prevented by indomethacin, demonstrating a cause–effect relationship between PGE-2 and repopulation. In support of this, we observed that aspirin strongly affects re-growth, though allowing for a weak repopulation rate, probably because of the residual PGE-2 present in the medium; aspirin action is however sufficient to prevent the acquisition of resistance to a second cycle of etoposide (Figure 2D).

Summarizing, etoposide-induced caspase-3 activation is responsible for PGE-2 production, which in turn is responsible for repopulation. The strict dependence of repopulation on the caspase-3/COX-2/PGE-2 axis, demonstrates that the etoposide-induced repopulation in PC3 occurs via Phoenix Rising.

### 2.3. XIAP Inhibition Potentiates the Phoenix Rising Pathway

**Figure 3:**
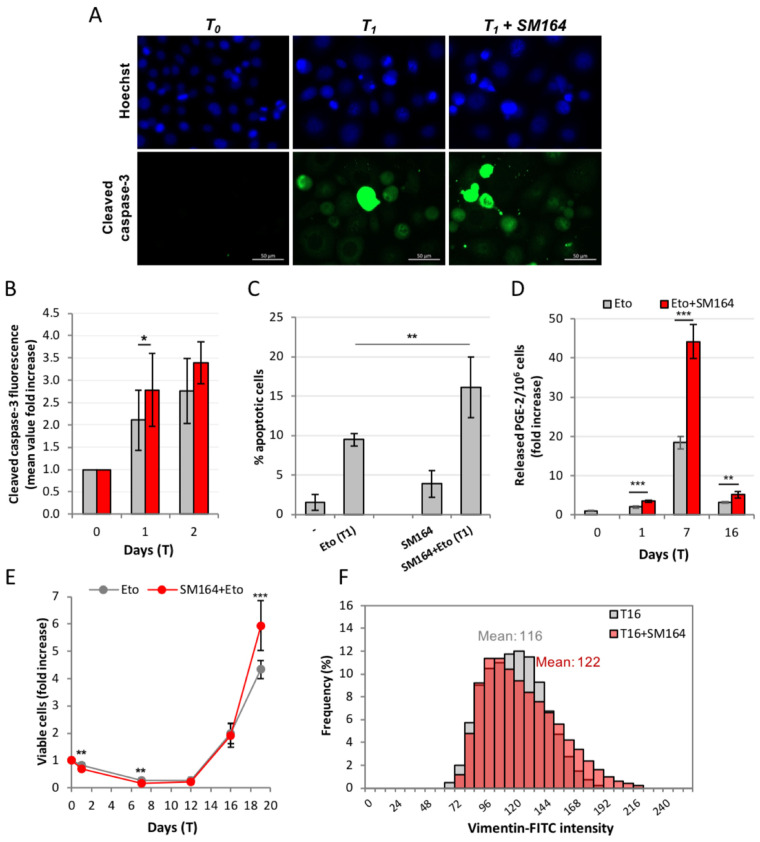
**XIAP inhibition potentiates the Phoenix Rising pathway.** XIAP inhibition with SM-164 increases etoposide-promoted caspase-3 activation: immunofluorescence with anti-cleaved caspase-3 antibody in wide-field micrography, where positive signals correspond to apoptotic cells (**A**); cleaved caspase-3 is quantified by flow cytometry (**B**); (**C**) SM-164 potentiates the pro-apoptotic effect of etoposide: the fraction of apoptotic cells was quantified by nuclear morphology, as described; (**D**) XIAP inhibition increases PGE-2 release: PGE-2 was quantified as previously described; (**E**) XIAP inhibition increases apoptosis (T_1_-T_12_) but later increases repopulation: cell number is quantified as previously described. Values are the mean of ≥3 independent experiments ± SD. Statistical significance was calculated via Student’s T test. * *p* < 0.05, ** *p* < 0.01 and *** *p* < 0.001 refer to the eto group; (**F**) XIAP inhibition increases vimentin expression in the repopulating cells: quantification of IF images of vimentin in cells repopulating from Eto (grey) and Eto+SM164 (red). The mean value is reported above each intensity distribution. The geomean and median values are the following: 114, 116 for T_16_; 119, 120 for T_16_ + SM164.

PC3 overexpress the anti-apoptotic protein XIAP, as many prostate cancer cells do [24], thus reducing their propensity to undergo apoptosis. We inhibited XIAP with the SMAC-mimetic small molecule SM-164 and observed that, as expected, this potentiates etoposide-induced caspase-3 activation (Figure 3A,B) and apoptosis (Figure 3C).

However, SM-164 also significantly increases PGE-2 production at all time points (Figure 3D), confirming the cause–effect relationship between caspase-3 activity and PGE-2 production. This is accompanied by reinforced repopulation, as shown in Figure 3E. All this shows that caspase-3 reactivation achieved by XIAP inhibition potentiates the Phoenix Rising pathway. Notably, SM-164 further increases the etoposide-induced high vimentin levels in Figure 3F, suggesting that the strength of the PGE-2 signal may modulate the fate of repopulating cells.

Overall, these results confirm that the post-etoposide repopulation in PC3 occurs via Phoenix Rising. The pro-apoptotic effect of SM-164 in etoposide-treated cultures, which is evident during the degenerative phase, turns into a pro-tumor effect during the repopulation phase, increasing cell proliferation and malignancy. The net effect of caspase reactivation seems, therefore, paradoxically, pro-tumoral.

### 2.4. PC3 Post-Therapy Repopulation Requires Epigenetic Reprogramming

To assess whether epigenetic cell reprogramming may play a role in repopulation, we used two inhibitors of the epigenetic processes acting on two different processes, i.e., histone de-acetylation or DNA methylation.

**Figure 4:**
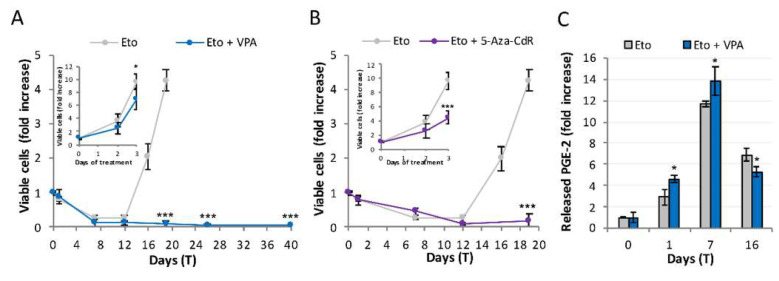
**CRAC requires epigenetic reprogramming.** (**A**) The HDAC inhibitor valproic acid (VPA) prevents etoposide-induced repopulation: the kinetics of viable (trypan blue-excluding) cell number in the presence/absence of valproic acid. VPA was added at T_0_ and re-administered at each medium change. Inset: VPA poorly affects untreated PC3 growth rate: results are expressed as fold increase over T_0_ (grey: control; blue: VPA); (**B**) The DNMT inhibitor 5-aza-2′-deoxycytidine (5-aza-CdR) prevents repopulation in etoposide-treated PC3: kinetics of viable (trypan blue-excluding) cell number upon etoposide insult in the presence/absence of 5-aza-2′-deoxycytidine. Inset: 5-aza 2′-deoxycytidine reduces, but does not impede, PC3 growth (grey: control; purple: 5-aza-CdR): results are expressed as fold increase over T_0_; (**C**) VPA increases PGE-2 production without promoting repopulation: time-course of PGE-2 production in PC3 treated with etoposide and valproic acid. The concentration of released PGE-2 is expressed as fold increase with respect to control values. Values are the mean of ≥ 3 independent experiments ± SD. Statistical significance was calculated via Student’s T test. * *p* < 0.05 and *** *p* < 0.001 refer to the eto group.

Valproic acid (VPA) is a protein de-acetylase inhibitor widely used to probe epigenetic rewiring, histones being preferred targets of the compound [25]. Figure 4A shows that 1 mM valproic acid, *per se* only slightly affecting untreated cells proliferation (inset), totally blocks repopulation, suggesting that PC3 need to undergo epigenetic reprogramming to repopulate after etoposide treatment. Notably, cells surviving etoposide treatment in the presence of VPA become senescent developing, and never losing, the beta-galactosidase signal.

To confirm that repopulation requires epigenetic reprogramming, we repeated the above-described experiment using the specific DNA methylation inhibitor 5-aza-2′-deoxycytidine, a base analog incorporated into DNA during replication, used at sub-toxic doses; notably, a pre-treatment is necessary here to allow for incorporation (see materials and methods). Figure 4B shows that 5-aza-2′-deoxycytidine, partially reducing proliferation in untreated PC3 (inset), strongly impairs repopulation. This confirms the requirement of epigenetic reprogramming in order to repopulate.

The lipidomic analysis shows that VPA does not impede PGE-2 production, which is even increased (Figure 4C), possibly due to an increased apoptosis rate occurring in the presence of VPA.

These data show that in the presence of VPA, Phoenix Rising is engaged and progresses up to PGE-2 production, i.e., the signal emission phase; however, it cannot proceed further, indicating that the passage to the successive steps, i.e., those related to signal perception by the target cells, requires epigenetic reprogramming.

### 2.5. Non-Tumor RWPE-1 Prostate Cells Undergo Phoenix Rising without Reprogramming or EMT

Phoenix Rising, originally described as a regenerating, wound-healing mechanism in normal tissues, is not typically a tumor-initiating mechanism. However, we just showed that in PC3 Phoenix Rising is strictly related to increased malignancy. We therefore explore here the eventual differences in Phoenix Rising between the etoposide-treated normal prostate cells RWPE-1, and PC3.

**Figure 5:**
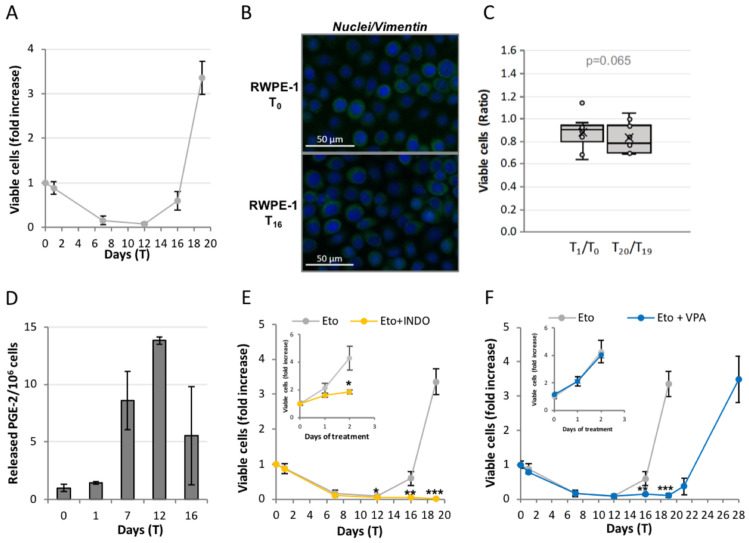
**Non-tumor RWPE-1 prostate cells undergo Phoenix Rising without epigenetic reprogramming.** (**A**) RWPE-1 cells were treated with 50 µM etoposide for 24 h and allowed to recover for 18 days. The profile shows the kinetics of viable (trypan blue-excluding) cell number expressed as fold increase over T_0_. The reported values are the mean of ≥ 3 independent experiments ± SD; (**B**) RWPE-1 repopulating cells do not undergo epithelial-to-mesenchymal transition, showing low and stable levels of the EMT-marker vimentin: fluorescence microscope images; (**C**) RWPE-1 do not acquire chemoresistance: ratio between cell number before (T_0_ and T_19_) and after (T_1_ and T_20_) the first vs. second etoposide treatment. There is no statistical difference (calculated via Mann–Whitney test) between the two ratio values; (**D**,**E**) Etoposide-treated RWPE-1 repopulate via the Phoenix Rising pathway: time-course of PGE-2 production (**D**) and kinetics of viable (trypan blue-excluding) cell number in the presence/absence of the COX-2 inhibitor indomethacin (INDO) (**E**). Inset: INDO reduces but does not prevent the proliferation of untreated RWPE-1 (gray: untreated; yellow: INDO). Results are expressed as fold increase over T_0_; (**F**) The HDAC inhibitor valproic acid (VPA) does not impede the etoposide-induced repopulation in RWPE-1: kinetics of viable (trypan blue-excluding) cell number in the presence/absence of VPA. VPA was added at T_0_ and re-administered at each medium change. Inset: VPA does not affect the proliferation of untreated RWPE-1 (gray: untreated; blue: VPA): kinetics of viable (trypan blue-excluding) untreated RWPE-1 in the presence/absence of VPA. The results are expressed as fold increase over T_0_. The reported values (E-F) are the mean of ≥ 3 independent experiments ± SD. Statistical significance was calculated via a Student’s T test. * *p* < 0.05, ** *p* < 0.01 and *** *p* < 0.001 refer to Eto or control group.

After etoposide, RWPE-1 undergo abundant cell loss (Figure 5A) in an early phase, after which they begin repopulating. Here, however, repopulation starts from a large number of surviving cells, as though a substantial fraction of the surviving cells is committed to repopulation. This strongly differs from what occurs in PC3, where only < 0.03% of the survivors are committed. RWPE-1 have a low basal level of vimentin, which is not changed in repopulating cells (Figure 5B), indicating that they do not undergo EMT; importantly, RWPE-1 do not develop resistance to a second etoposide treatment (Figure 5C). PGE-2 levels also increase in RWPE-1 upon etoposide treatment (Figure 5D), and COX inhibition with indomethacin totally inhibits repopulation (Figure 5E): this indicates that etoposide-induced repopulation occurs via Phoenix Rising also in these cells. However, valproic acid here does not inhibit repopulation (Figure 5F).

These results show that in RWPE-1, Phoenix Rising is elicited upon etoposide treatment, but it does not imply EMT, nor does it require epigenetic reprogramming. This may constitute a key point explaining the different effects of Phoenix Rising in normal vs. cancer tissues.

### 2.6. Etoposide-Treated LNCaP Cells Activate but Do Not Complete the Phoenix Rising Pathway

LNCaP cells originate from a node metastasis of a milder prostate cancer type, being sensitive to androgen stimulation [26]. Although androgen-sensitive PCa patients are not typically treated with chemotherapy, we explored the repopulation issue in a system of androgen sensitive PCa as a comparison with the castration-resistant type.

**Figure 6:**
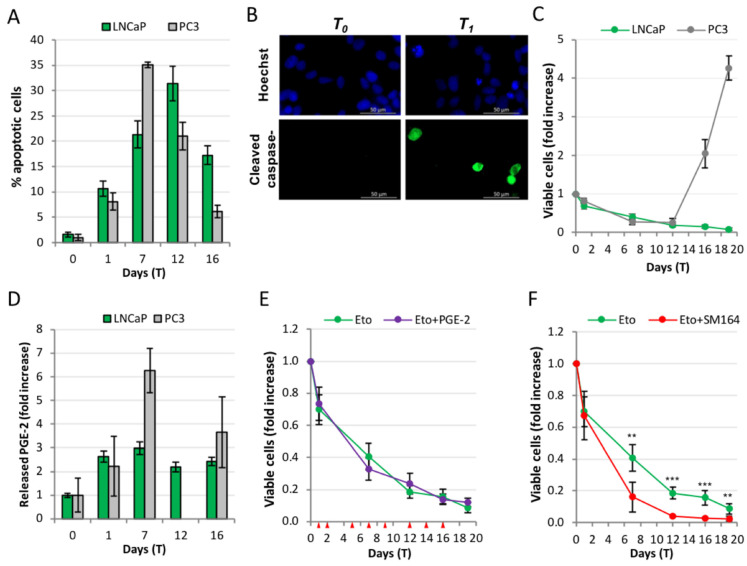
**AR+ LNCaP PCa cells do not activate the Phoenix Rising pathway.** (**A**) Time course of etoposide-induced apoptosis in PC3 vs. LNCaP; apoptotic cells were quantified by nuclear morphology as described; (**B**) Immunofluorescence with anti-cleaved caspase-3 antibody of untreated (T_0_) or etoposide-treated (at T_1_) cells; positive signals correspond to apoptotic cells, showing that LNCaP undergo caspase-3-mediated apoptosis; (**C**) Comparison of the kinetics of viable cells in PC3 vs. LNCaP cells treated with etoposide, normalized over T_0_ values for each cell line: LNCaP do not repopulate; (**D**) Released PGE-2 in etoposide-treated PC3 vs. LNCaP, expressed as fold increase over each control, showing that both cells upregulate PGE-2 levels over the respective controls; (**E**) addition of exogenous PGE-2 fails to promote CRAC in LNCaP; PGE-2 was administered at the indicated time points (see red triangles) at the following concentrations: 9 (T_0_), 10 (T_1_), 11 (T_2_), 15 (T_5_), 20 (T_7_), 21 (T_9_), 25 (T_12_), 27 (T_14_), 30 (T_16_) and 34 (T_19_) nM; cell number is expressed as fold increase over T_0_; (**F**) The XIAP inhibitor SM-164 sensitizes to apoptosis (T_1_-T_12_): cell number is expressed as fold increase over T_0_. The reported values are the mean of ≥3 independent experiments ± SD. Statistical significance was calculated via Student’s T test. ** *p* < 0.01 and *** *p* < 0.001 refer to the eto group.

Figure 6A shows the time course of apoptosis in LNCaP compared with that of PC3 cells; in both cell lines the apoptosis rate slowly increases, reaches a peak and subsequently decreases; peaks occur at different time points, i.e., T_7_ for PC3 and T_12_ for LNCaP. Apoptosis is characterized by high expression of cleaved caspase-3, indicating a canonical process (Figure 6B). Figure 6C shows the kinetics of cells recovering from 24 h of etoposide treatment. Strikingly, and unlike PC3, in LNCaP the degenerative apoptotic phase is not followed by repopulation, and the culture slowly evolves toward extinction. Etoposide causes LNCaP to develop senescence, as shown by the beta-galactosidase signal; importantly, the signal does not disappear for the whole period of the experiment (data not shown).

The PGE-2 level increases also in LNCaP, reaching an approximate 2.5-fold increase throughout the experiment. Importantly, adding exogenous PGE-2, taking care of mimicking the levels spontaneously reached in treated PC3, did not restore repopulation (Figure 6E). It is worth noting that in the COX inhibition/reconstitution experiments performed in PC3, PGE-2 addition restores repopulation when it is prevented by COX inhibitors (work in preparation), implying that an exogenous PGE-2 administration can be effective if the cells are sensitive. This indicates that the pro-repopulation signaling downstream of PGE-2 is impaired in LNCaP.

We then explored the effect of SM-164, considering that LNCaP also over-express XIAP [24]. In this case as well, XIAP inhibition sensitizes to apoptosis, whose values increase from 1.55 ± 0.51% to 10.6 ± 1.53% at 24 h of 50 µM etoposide, reaching 14.11 ± 0.84% in the presence of SM-164. Figure 6F shows that XIAP inhibition strongly increments the rate of the etoposide-induced cell loss; in the absence of repopulation, this loss is not counterbalanced, and the net result is an acceleration of culture extinction, i.e., a net anti-cancer effect.

LNCaP thus represent a cell cancer model refractory to Phoenix Rising in spite of caspase-3 activation and PGE-2 production, suggesting that the signaling axis is interrupted, possibly at the level of PGE-2 signal perception or processing.

## 3. Discussion

We report here that a simple, purely epithelial in vitro model of PCa cells treated with etoposide effectively reproduces the complexity of a wounded-tissue response to damage. Monitoring not only the degenerative, but also the repopulation phase, we observed that resistance acquisition develops via adaptive mechanisms, which allow for the reprogramming of a tiny fraction of surviving cells. This originates regeneration foci of next-generation cells, displaying increased chemo-resistance. 

The ability to elicit such effects is not exclusive of etoposide, but is shared by all pro-apoptotic chemotherapeutic agents tested (e.g., spindle poisons or DNA alkylating agents, work in preparation), showing it does not depend on the mechanism of action of the drug, but rather on a common cell and tissue response. Remarkably, apoptosis seems to be the driver of repopulation, which occurs via Phoenix Rising.

Cancer relapse after therapy-induced remission is a major clinical issue, but the mechanisms through which this occurs are still largely unknown; establishing a reliable in vitro model where to observe and experimentally modulate the initial events of post-therapy repopulation would greatly help understanding and even controlling the process. The published in vitro studies of Phoenix Rising make generally use of a “two-pot” approach, where irradiated or chemo-treated feeder cells (which may be either stroma fibroblasts or cancer cells) are placed in a trans-well co-culture with untreated recipient cancer cells, to stimulate replication in the latter [17,27,28]; since recipient cells are already in a state of proliferation, the measured effect is just an increase in the proliferation rate. Such studies clarified the initial steps of Phoenix Rising. To study the role of Phoenix Rising in repopulation, however, a different experimental strategy is required. Indeed, in tissues of treated patients, the cells that repopulate and give rise to relapse were subjected to therapy, experiencing stress signals, DNA damage response [29], entering senescence and becoming quiescent; only eventually they repopulate, and to do so specific signaling events are required [15,21]. Therefore, untreated cells are not the right target for the repopulation signals. A major objective of our study was to set up a straightforward, self-sufficient in vitro model of therapy-induced cancer repopulation. To this purpose, we established a “one-pot” approach, demonstrating that the treated cancer cells can be both producers and recipients of the repopulation paracrine secretions. The “trick” we envisaged was letting cells self-organize after the strong apoptotic phase, during which they assume novel, dramatically different morphologies. Such cells condition the environment, suggesting that the emission and reception of the paracrine secretion may be taken care of by different cells acquiring novel functions. This is consistent with epithelial cells playing non-canonical roles, as a surrogate of those usually played by other cellular components [30,31,32], e.g., stroma, here absent. These activities allowed cells to develop a kind of multi-faceted pseudo-cancer microenvironment, able to promote cell reprogramming, EMT and repopulation with cells that deeply differ from the original, stably displaying resistance to a second treatment. These effects faithfully resemble what occurs in treated patients: this experimental strategy is in fact a breakthrough tool allowing modeling cancer relapse in vitro.

The main goal of our one-pot model was to explore the relationship between the different tissue-defense pathways elicited by chemotherapy, namely compensatory proliferation and epigenetic reprogramming, which are major limitations to the success of cytotoxic pulsed treatments. We used as a model system the prostate cancer cell PC3, with the non-tumor prostate RWPE-1 as a healthy control. In RWPE-1, post-etoposide repopulation occurs through Phoenix Rising, does not require epigenetic reprogramming, and the repopulating cells resemble the originals. This confirms what emerges from the abundant literature on Phoenix Rising, which shows that in many organisms this pathway allows wounded tissues to recover and restore a normal homeostasis [17,19]. By contrast, PC3 prostate cancer cells’ repopulation, though following Phoenix Rising, is accompanied by EMT, acquired chemo-resistance and requires epigenetic rearrangement. In particular, the histone de-acetylase inhibitor valproic acid interrupts the Phoenix Rising pathway after the step of PGE-2 production, totally inhibiting repopulation. This indicates that up to that point, that is, as far as the emission of proliferation signals is concerned, the pathway does not require epigenetic rearrangement. Instead, rearrangement is required for reception or processing of the proliferation signals by the target cells. This means that Phoenix Rising in cancer cells needs to be “assisted” by the epigenetic rearrangement machinery in order to be completed. This also implies the simultaneous occurrence of EMT and acquired resistance; therefore, Phoenix Rising intimately participates in tumor progression. Cancer repopulation and acquired cell resistance are thus coupled in therapy-promoted tumor progression, possibly constituting a complex and branched signaling package that may be convenient to consider as a unique process (CRAC). Accordingly, recent papers show that in bladder cancer PGE-2-mediated repopulation is correlated to increased cell resistance [33] and tumor progression [15]. 

DNA damage produces a halt in cell proliferation in order to repair the lesions, after which the cells that were successfully repaired become committed to resuming replication. Here, we observed that commitment to repopulate is achieved in different ways in RWPE-1 vs. PC3. It is quite straightforward in the former, where many surviving cells resume growing, suggesting a prompt repair in a substantial fraction of the cells. Instead, the process is troublesome in the latter: indeed, only a very tiny fraction of surviving PC3 achieves the task, and only upon reprogramming, suggesting an impaired DNA repair. It is well described that cells unable to swiftly and fully repair DNA damage enter a “premature senescent” status, implying a stable inhibition of proliferation [34]. However, cancer cells can escape from senescence through epigenetic reprogramming [35], gaining malignancy [36,37]; this constitutes a concern, and senolytic treatments have been proposed as a way to limit therapy-induced senescence [38]. Escape from senescence relates especially with those cancer cells lacking key tumor suppressor gene expression, such as TP53 [39]. Notably, by modulating the expression of a TRP53 transgene through an inducible promoter, it was shown that escape from senescence greatly favors the acquisition of malignant traits [40]. Normal cells, though able to undergo premature senescence in response to DNA damage or oncogenic stress, typically do not escape, permanently remaining in a metabolically active, replication-inactive state [34]. In our system PC3, which are TP53-null, undergo senescence upon etoposide treatment, while the reprogrammed, repopulating cells become beta-galactosidase-negative: this suggests that PC3 repopulating from etoposide treatment may acquire the highly malignant traits by entering in, and successively escaping from, senescence. Intriguingly, PC3 surviving etoposide treatment in the presence of valproic acid develop, and never lose, a high beta-galactosidase signal: this suggests that the epigenetic rearrangements inhibited by valproic acid may include those necessary for escaping from senescence. 

LNCaP do not repopulate after etoposide treatment, though increasing PGE-2 levels at all time points; this is reminiscent of what occurs in PC3 in the presence of valproic acid, suggesting that in LNCaP Phoenix Rising cannot be completed due to an intrinsic inability to undergo epigenetic reprogramming after DNA damage. Interestingly, it is reported that in prostate cancer, p53 stabilizes the senescence status, suppressing cancer cell plasticity [41]. It is tempting to speculate that the TP53^wt/wt^ LNCaP cells, which readily undergo senescence upon etoposide treatment, may be unable to repopulate because p53 prevents reprogramming and escape from senescence. In support of this, we found that therapy-promoted p53 silencing enables LNCaP to repopulate and acquire malignant traits upon etoposide treatment (work in preparation). This observation implies that the role of epigenetic plasticity in repopulation, and its dependence on p53 expression, may be explored as a possible predictive factor for a positive response to cytotoxic therapies. 

The presented in vitro data bring into focus that tumor progression is a homeostatic, dynamic process which can be separated in different steps, and could be therapeutically targeted. Tumor type-dependent CRAC might in fact be a major reason for treatment failure. Clinically, some tumors show a typically weak tendency to repopulate after chemotherapy [42]; in other tumors, seldom and largely unpredictably, chemotherapy may lead to continuous complete remission [43]. Overall, however, most pro-apoptotic treatments in metastatic disease are in fact palliative [44,45]. This is generally attributed to tumor heterogeneity and genetic-based resistance [46,47]; our evidence, however, indicates CRAC development as an additional reason for pro-apoptotic therapy failure. Based on our data, it would be important to understand the biologic reasons of the different response, to determine which tumors and which patients may really benefit from apoptosis-inducing therapies. 

Apoptosis is still a major objective of anticancer therapies, leading to effective tumor reduction. However, the phenomenon of compensatory proliferation, and the overlapping of cancer repopulation with acquired cell resistance, raises concerns about the common use of pro-apoptotic therapies, identifying precise intrinsic limits in the fact that the wounded cancer tissues actively setup responses aimed at tissue-defense. Apoptosis thus becomes a double-faced process in anticancer therapies, which provides initial benefits that may later turn into Trojan horse [18,21]. Targeting anti-apoptotic proteins, often overexpressed in cancer as a mean of cell-resistance, translational studies proved an efficient way of sensitizing to apoptosis; however, when translated into clinical trials, the results were controversial, at least for solid tumors [48,49], suggesting that additional factors determine the final effect in the long run. We provide here evidence that the inhibition of the anti-apoptotic protein XIAP potentiates etoposide-induced apoptosis in an initial phase. However, extending the time of observation, this eventually turns into enhanced repopulation, by reinforcing the Phoenix Rising pathway, outweighing the initial benefits. Intriguingly, in etoposide-treated LNCaP, which fail to repopulate, XIAP inhibition increments etoposide-induced apoptosis without successive counter-effects, possibly benefiting the final outcome. This suggests that the outcome of XIAP-targeting chemo-strategies in particular, and pro-apoptotic therapies in general, may depend on the cells’ propensity to repopulate. 

Recently, anakoinosis was proposed as a successful way of modulating cancer tissue homeostasis by re-establishing a new equilibrium via “tissue editing” [50]. Pro-anakoinotic drugs act as tissue master modulators, thereby preventing tissue defenses, providing a concerted regulative activity profile of a treatment schedule’s single components, and may facilitate long-term tumor control or even continuous complete remission [51,52,53,54]. Importantly, this approach showed that classic pro-apoptotic drugs are un-necessary to induce long-term tumor control or continuous complete remission [55]: anakoinosis would thus bypass the intrinsic limitation of the current pro-apoptotic therapies by aiming at bio-modulation instead of cytotoxicity, possibly avoiding CRAC as suggested by *in silico* modeling [56]. 

The notion that chemotherapy-promoted resistance is not only due to selection of the most aggressive oncogene mutations, but requires paracrine stimulation (e.g., PGE-2 signaling) and epigenetic rearrangement [57], implies that therapy-promoted CRAC is a druggable process. Clinical trials targeting PGE-2 production [33] or caspase-3 [58] are indeed being proposed, with promising prospective applications. Associating agents that modulate Phoenix Rising or epigenetic reprogramming, with conventional pulsed chemotherapeutic drugs, during the time window when surviving cells are amenable for commitment to repopulation, might stabilize a positive response, preventing or at least delaying relapse. Therefore, a CRAC-free chemotherapy would be achievable in principle, thereby taking advantage of the potent tumor-reducing effect of the pro-apoptotic approaches, without their important adverse, pro-relapse effects.

## 4. Conclusions

**Figure 7:**
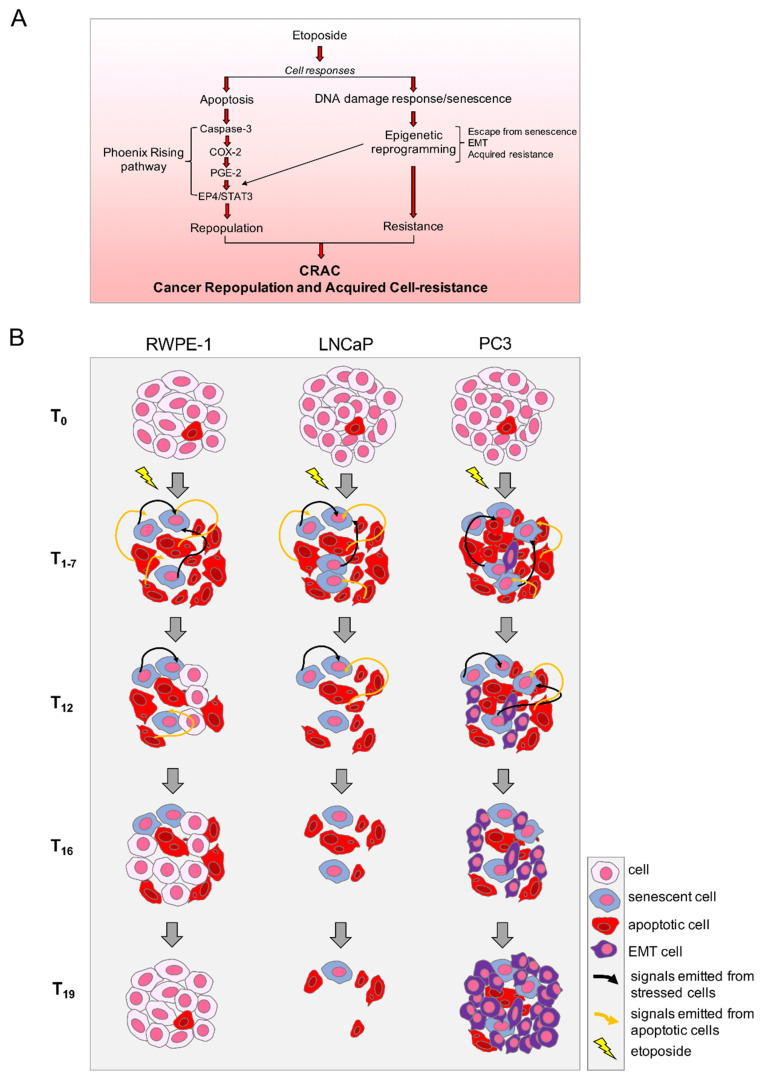
**Therapy-induced cancer repopulation and acquired cell-resistance (CRAC) on prostate tumor cells.** (**A**) Etoposide-induced cell signaling pathways promoted by apoptotic cells (left) and surviving stressed cells (right) converge into cancer repopulation and acquired cell-resistance (CRAC); (**B**) Evolution of the etoposide-treated cultures leading to different outcomes on the different cell types tested.

The picture emerging from in vitro CRAC modeling in prostate cancer systems treated with etoposide, is suggestive of a process that requires specific paracrine signaling and cell response pathways, as schematized in Figure 7A. Our preliminary evidence that other pro-apoptotic anticancer drugs exert similar effects on PC3, suggests that such signaling results from a convergence of the specific damage-induced responses into a general reaction, such as, e.g., the integrated stress response [59]. It remains to be explored what happens in other cancer types; however, phenomena such as EMT and PGE-2-mediated inflammation, as well as the occurrence of Phoenix Rising, are widely occurring in response to therapy in cancers such as bladder, colorectal, pancreatic, breast, oropharyngeal, etc. [33,60,61,62,63,64], allowing hypothesizing that CRAC may be a general occurrence.

Rather, a difference may be present between cancers belonging to the same histotype, as suggested by the different behavior of LNCaP and PC3 presented here. Indeed, the different responses of the two prostate cancer cell lines, schematized in Figure 7B, suggest that the outcome of pro-apoptotic therapies may depend on the propensity of damaged cells to repopulate, possibly related to their genetic asset: the aggressive, castration-resistant p53null PC3 react to etoposide repopulating with increased malignancy, whereas the milder androgen-sensitive, p53wt LNCaP attempt Phoenix Rising but fail to compete the pathway and repopulate.

- non-tumor RWPE-1: etoposide promotes cell death, or stable senescence (p53^+/+^), or repair. Repaired cells repopulate with features identical to the originals;
- androgen-sensitive tumor LNCaP: etoposide promotes cell death or stable senescence (p53^+/+^); repair looks non-performant; no repopulation;
- castration-resistant tumor PC3: etoposide promotes cell death or unstable senescence (p53 null); repair looks non-performant; few scattered surviving cells undergo epigenetic reprogramming, possibly escaping from senescence and resuming proliferation so as to repopulate the depleted culture; repopulating cells acquire EMT and resistance to a second etoposide treatment.

These novel viewpoints may have unexpected applicative potentials in the clinic, aiming at achieving a CRAC-free chemotherapy.

On one side, a thorough survey of the genes and environmental mechanisms involved in such responses may help setting up a screening protocol aimed at identifying patients that may positively react to pro-apoptotic therapy with no or weak repopulation.

On the other, CRAC might be therapeutically targeted by a focused approach in addition to apoptosis inducing therapies. Interestingly, tumor tissue remodeling following pro-anakoinotic therapy approaches may inhibit tumor cell repopulation, as indicated by long-term disease stabilization after therapy discontinuation, as shown for prostate cancer and multiple myeloma [50,65]. The results obtained with anti-CRAC agents such as inhibitors of epigenetic reprogramming or PGE-2 production, suggest that the competence to repopulate is limited to a defined time-frame after apoptosis induction, after which repopulation cannot occur any longer, or occurs without increased malignancy. This provides the logical basis to pharmacologically dissociate apoptosis-induced tumor reduction from relapse. Combining the standard therapy with a brief treatment with modulatory agents that delay repopulation would open an efficient therapeutic option also for patients with cancers prone to repopulate, stabilizing the disease or at least delaying relapse.

## 5. Materials and Methods

### 5.1. Cell Culture

Human prostate cancer PC-3 and LNCaP cells (ATCC) were grown in RPMI 1640 medium supplemented with 10% fetal bovine serum (FBS), 100,000 units/L penicillin, 50 mg/L streptomycin and 200 mM glutamine (Euroclone). Non-neoplastic, immortalized human prostatic epithelial cells, RWPE-1 (ATCC), were grown in keratinocyte serum-free medium (K-SFM), supplemented with 1% penicillin/streptomycin (100 IU/mL), 50 μg/mL bovine pituitary extract and 5 ng/mL epidermal growth factor (Life Technologies). All cells were grown at 37 °C in a humidified atmosphere of 5% CO_2_ in air and routinely split by trypsinization with Trypsin-EDTA (Euroclone). Experiments are performed with a base-viability >98%.

### 5.2. Treatments

Apoptosis induction: the topoisomerase II inhibitor etoposide (Sigma-Aldrich) was used at the final concentration of 50 µM and washed out after 24 h. Second treatments were performed at T_16_ or T_19_, as specified, for 24 h. XIAP inhibition: the SMAC mimetic SM-164 (kindly provided by Dr. Shaomeng Wang, University of Michigan Comprehensive Cancer Center, Ann Arbor, MI, USA) was administered 1 h prior to the etoposide insult at the final concentration of 10 nM. Caspase inhibition: caspase-3 inhibitor Z-DEVD-fmk (Calbiochem, San Diego, CA, USA) and pan caspase-inhibitor Z-VAD-fmk (Enzo Life Sciences, Farmingdale, NY, USA) were used at the final concentrations of 10 µM and 2 µM, respectively, and added 1 h before etoposide. COX-2 inhibition: 100 µM Indomethacin (Sigma-Aldrich, St. Louis, MO, USA) or 1 mM Aspirin (Cayman Chemical, Ann Arbor, MI, USA) were added together with etoposide and kept throughout the CRAC protocol. HDAC inhibition: 1 mM Valproic acid (Sigma Aldrich, St. Louis, MO, USA) was added together with etoposide and kept throughout the CRAC protocol. DNMT inhibition: 5-aza-2′-deoxycytidine (Sigma Aldrich, St. Louis, MO, USA) at the final concentration of 1 µM was administered for two weeks as pre-treatment.

PGE-2 supplementation: PGE-2 (Cayman Chemical, Ann Arbor, MI, USA) was added at the concentrations and time points indicated in the legends.

Probes and reagents: Hoechst 33342, DAPI and all the other chemicals used for the clonogenic assay were purchased from Sigma-Aldrich (St. Louis, MO, USA).

### 5.3. CRAC Protocol

PC-3 cells were seeded at a cell density of 12,500/cm^2^; RWPE-1 and LNCaP, instead, were seeded at a cell density of 17,500/cm^2^. At 48 h post-seeding, cells were treated with etoposide for 24 h, washed out and allowed to recover for 18 days, changing the medium as indicated (red triangles). A second etoposide cycle was administered at day 16 or 19, as indicated. Viable cell number was evaluated at selected time points following trypsinization and assessed using a Burker counting chamber by the Trypan Blue exclusion method.

Determination of cell-resistance: effect of the first (T_0_) vs. the second (T_16_ or T_19_) etoposide treatments was compared by determining the ratio between cell number after 24 h of etoposide treatments (i.e., T_1_, T_17_ or T_20_) and before treatment (i.e., T_0_, T_16_ or T_19_, respectively). The higher the value, the lower the efficacy of the etoposide treatment.

### 5.4. Quantification of Apoptosis

Apoptosis was evaluated quantifying the fraction of apoptotic nuclei by fluorescence microscopy (ZEISS Axio Observer) after DNA staining with the cell-permeable specific dye Hoechst 33342 at the final concentration of 10 μg/mL, directly added to the cell culture. The fraction of apoptotic nuclei among the total cell population was calculated by counting 300 cells in at least three independent, randomly selected microscopic fields [66,67].

### 5.5. Senescence Beta-Galactosidase Staining

Senescence of etoposide-treated PC3 cells was assessed through the senescence beta-galactosidase staining kit (Cell Signaling, Danvers, MA, USA). Briefly, cells grown in 6-well plates were washed with PBS, fixed for 15 min and stained with the beta-galactosidase staining solution overnight at 37 °C in a dry incubator. Samples were then checked under a light transmission microscope (200× total magnification) for the assessment of blue color.

### 5.6. Clonogenic Assay

At the indicated time points, cells were trypsinized, and 1000 cells for each sample were seeded in 100-mm tissue culture dishes in triplicate. Colonies were allowed to form for 10 days, then fixed with methanol/acetic acid (7:1), stained with crystal violet and quantified by visual inspection. Colonies with >80 cells were scored.

### 5.7. Immunofluorescence

#### 5.7.1. Cleaved Caspase-3 Levels (Flow Cytometry) 

Cells treated with etoposide ± SM-164 were trypsinized, washed by centrifugation with PBS, fixed with 4% paraformaldehyde for 15 min, washed with PBS and permeabilized with ice-cold 90% methanol for 30 min on ice. Washed samples were then incubated with primary antibody against cleaved caspase-3 (Cell Signaling, Danvers, MA, USA) for 2 h at room temperature. After three washes in PBS, cells were subjected to FITC-conjugated secondary antibody (Sigma-Aldrich, St. Louis, MO, USA) at room temperature for 1 h. A total of 10,000 cells for each sample was analyzed by FACSCalibur flow cytometer. Data were analyzed using Flowing software.

#### 5.7.2. Cleaved Caspase-3 (Fluorescence Microscopy)

Samples grown over Nunc Lab-Tek chambers (Thermo Fisher, Waltham, MA, USA) were washed with PBS, fixed with 4% paraformaldehyde for 15 min, washed three times with PBS and blocked with 1% BSA for 60 min. Samples were then incubated with primary antibody against cleaved caspase-3 (Cell Signaling, Danvers, MA, USA) in PBS 0.3% Triton 1% BSA overnight at 4 °C. After three washes with PBS, the samples were incubated with the FITC-conjugated secondary antibody (Sigma-Aldrich, St. Louis, MO, USA) at room temperature for 1 h. The samples were washed three times with PBS, and the cell nuclei were finally stained with Hoechst 33342 (10 µg/mL) for 15 min at room temperature. Images were captured using a ZEISS Axio Observer microscope and analyzed through the Carl Zeiss Microscopy GmbH’s ZEN software.

#### 5.7.3. Vimentin/β-Catenin Detection by Fluorescence Microscopy

Samples grown over Nunc Lab-Tek chambers (Thermo Fisher) were washed with PBS, fixed with 4% paraformaldehyde for 15 min, washed three times with PBS, permeabilized in PBS 0.3% Triton for 10 min and blocked with 1% BSA for 30 min. The samples were then incubated with primary antibody against vimentin (Sigma-Aldrich, St. Louis, MO, USA) or β-catenin (Ventana, Oro Valley, AZ, USA) in PBS 1% BSA for 1 h at room temperature. After three washes with PBS, the samples were incubated with the FITC-conjugated (vimentin) or TRITC-conjugated (β-catenin) secondary antibody (Sigma-Aldrich, St. Louis, MO, USA) at room temperature for 1 h. The samples were washed three times with PBS, and the cell nuclei were finally stained with DAPI (2 μg/mL). Images were captured using a ZEISS Axio Observer microscope; unbiased staining intensity was estimated through pixel fluorescence analysis according to the Carl Zeiss Microscopy GmbH’s ZEN software [68]. At least four images for each sample were analyzed to quantify vimentin intensity.

### 5.8. HPLC PGE-2 Measurement

The eicosanoid PGE-2 was measured in PC-3, RWPE-1 and LNCaP cell media by HPLC\MS\MS analysis. Measurements were performed using an Agilent Technologies 1200 HPLC Liquid Chromatography System coupled to an electrospray ionization (ESI) triple quadrupole 6400 mass spectrometer (Agilent Technologies, Santa Clara, CA, USA), and data acquisition was performed using Mass Hunter software. The instrument was operated in negative ionization mode. For the optimization of MS and MS/MS conditions, standards at a concentration of 10 ng/uL were introduced into the spectrometer by direct infusion through a syringe pump (flow rate of 10 μL/min) into the HPLC solvent flow (flow rate 0.2 mL/min). The capillary voltage was set at 3500 V, the desolvation temperature at 350 °C, the nebulizer gas at 20 psi and the cone voltage at 35 V. Eicosanoids were analyzed using scheduled multiple reaction monitoring (MRM) [69]. The collision energy was optimized using nitrogen as the collision gas. A chromatographic analysis was performed on a C18 column (Symmetry, 3.5 µm, 100 Å ~2.1 mm, Waters). The injection volume was 5 μL, and the flow rate 0.2 mL/min. The column was maintained at room temperature. The analysis was performed using a methanol-based isocratic system obtained by mixing two solvents (A and B) at a ratio of 95:5 (*v*/*v*). Solvent A was methanol/water/glacial acetic acid, 80:20:0.02 (*v*/*v*/*v*); solvent B was acetonitrile/water/glacial acetic acid, 45:55:0.02 (*v*/*v*/*v*). PGE-2 quantification was performed by the stable isotope dilution method: an internal standard was selected, and a linear curve was generated, where the ratio of the analyte standard peak area to the internal standard peak area was plotted versus the amount of the analyte standard.

### 5.9. Statistical Analysis

Each experiment was repeated ≥3 times. For data presentation, the mean ± SD was used for homogeneous objects; for dis-homogeneous objects distributed as a non-Gaussian population, the entire data distribution was reported. The latter is presented as boxplot, which displays the distribution of data based on five numbers (minimum, first quartile, median, third quartile and maximum), giving information on data symmetry, distortion and presence of outliers. The statistical evaluation was conducted by Student’s T Test (significance set at *p* < 0.05) or nonparametric Mann–Whitney test, using the Past 4.06b software.
